# Influence of dosing times on cisplatin-induced peripheral neuropathy in rats

**DOI:** 10.1186/s12885-016-2777-0

**Published:** 2016-09-27

**Authors:** Yoshihiro Seto, Fumiyasu Okazaki, Keiji Horikawa, Jing Zhang, Hitoshi Sasaki, Hideto To

**Affiliations:** 1 Department of Medical Pharmaceutics, Graduate School of Medicine and Pharmaceutical Sciences for Research, University of Toyama, 2630 Sugitani, Toyama, 930-0194 Japan; 2 Graduate School of Science and Engineering, University of Toyama, Toyama, Japan; 3 Hospital Pharmacy, Nagasaki University Hospital, Nagasaki, Japan

**Keywords:** Cisplatin, Peripheral neuropathy, Chronotherapy

## Abstract

**Background:**

Although *cis*-diamminedichloro-platinum (CDDP) exhibits strong therapeutic effects in cancer chemotherapy, its adverse effects such as peripheral neuropathy, nephropathy, and vomiting are dose-limiting factors. Previous studies reported that chronotherapy decreased CDDP-induced nephropathy and vomiting. In the present study, we investigated the influence of dosing times on CDDP-induced peripheral neuropathy in rats.

**Methods:**

CDDP (4 mg/kg) was administered intravenously at 5:00 or 17:00 every 7 days for 4 weeks to male Sprague–Dawley rats, and saline was given to the control group. To assess the dosing time dependency of peripheral neuropathy, von-Frey test and hot-plate test were performed.

**Results:**

In order to estimate hypoalgesia, the hot-plate test was performed in rats administered CDDP weekly for 4 weeks. On day 28, the withdrawal latency to thermal stimulation was significantly prolonged in the 17:00-treated group than in the control and 5:00-treated groups. When the von-Frey test was performed to assess mechanical allodynia, the withdrawal threshold was significantly lower in the 5:00 and 17:00-treated groups than in the control group on day 6 after the first CDDP dose. The 5:00-treated group maintained allodynia throughout the experiment with the repeated administration of CDDP, whereas the 17:00-treated group deteriorated from allodynia to hypoalgesia.

**Conclusions:**

It was revealed that the severe of CDDP-induced peripheral neuropathy was inhibited in the 5:00-treated group, whereas CDDP-treated groups exhibited mechanical allodynia. These results suggested that the selection of an optimal dosing time ameliorated CDDP-induced peripheral neuropathy.

**Electronic supplementary material:**

The online version of this article (doi:10.1186/s12885-016-2777-0) contains supplementary material, which is available to authorized users.

## Background

Cisplatin, *cis*-diamminedichloro-platinum (CDDP), is the first agent of platinum-based anticancer drugs, and acts by crosslinking DNA and inhibiting DNA replication [1, 2]. It is extensively used in the treatment of non-small cell lung, head and neck, ovarian, and breast cancers [3–6], and exhibits strong therapeutic effects in cancer chemotherapy. Although CDDP is the main drug used in chemotherapy, it has dose-limiting adverse effects such as peripheral neuropathy, nephropathy, and vomiting, which limit the continuation of chemotherapy [7–9]. As a countermeasure to CDDP-induced adverse effects, fluid therapy and diuretic drugs have been used for nephropathy [10, 11]. Serotonin 3 receptor antagonists and neurokinin-1 receptor antagonists have also been administered to prevent vomiting [12–16]. However, no effective treatment currently exists for peripheral neuropathy [17–20].

CDDP-induced peripheral neuropathy frequently occurs in patients receiving CDDP at a total dose of more than 300 mg/m^2^ [21]. Patients with CDDP-induced peripheral neuropathy have difficulty moving their arms, feet, and fingers as well as difficulty walking, numbness, dysesthesia, sensory abnormalities, and autonomic neuropathy [21–23]. The chronic symptoms of CDDP-induced neuropathy, which may continue for months after the cessation of treatment, may be due to the accumulation of platinum compounds in the dorsal root ganglia (DRG) [24, 25]. The quality of life (QOL) of patients with these symptoms is reduced and these adverse effects also limit the continuation of chemotherapy. Therefore, the prevention or suppressing of CDDP-induced peripheral neuropathy is desired.

The success of chronotherapy in ameliorating adverse effects and achieving improved therapeutic effects has been attributed to a deeper understanding of pathological characteristics and pharmacological characterization as well as the selection of an optimal dosing time for drug administration [26, 27]. Previous studies reported that chronotherapy was beneficial for the adverse effects of CDDP. Nephropathy was ameliorated when CDDP was administered in the active phase of these animals [28–31]. Moreover, vomiting induced by CDDP improved in patients with urogenital cancer when CDDP was administered at 17:00 [32]. Thus, chronotherapy may ameliorate CDDP-induced peripheral neuropathy.

In the present study, we investigated the influence of dosing times on CDDP-induced peripheral neuropathy in rats, and the pharmacokinetics of CDDP in serum and the DRGs in order to elucidate the mechanisms responsible for dosing time-dependent differences.

## Methods

### Animals

Male Sprague–Dawley (SD) rats (6 weeks old) were purchased from Japan SLC, Ltd. (Japan). Rats were housed two to three per cage under standardized light–dark cycle conditions (lights on and off at 7:00 and 19:00, respectively) at a room temperature of 23–25 °C and humidity of 50– 60 % with free access to food and water. All rats were kept under these conditions for 1 week until used in experiments, and the body weights were 220–260 g at point of starting experiments. Experiments were performed after formal approval by the Committee for Animal Experiments at the University of Toyama.

### Preparation of CDDP

CDDP, supplied by Nippon Kayaku Co., Ltd. (Tokyo, Japan), was dissolved in saline. Its final concentration was 2 mg/mL (4 mg/kg) in each dosing group. CDDP was intravenously (i.v.) administered at 2 mL/kg to rats.

### Experiment I: Influence of CDDP administration on peripheral neuropathy

CDDP was administered i.v. at 17:00 every 7 days for 4 weeks to rats (*n* = 4 in Hot plate test, *n* = 8 in von-Frey test). Saline was given to the control group (*n* = 4 in Hot plate test, *n* = 8 in von-Frey test).

The withdrawal threshold was measured on days −1, 6, 13, 20, and 27 using the von-Frey test during the administration of CDDP. Withdrawal latency by heat stimulation was determined on days −1, 6, and 27 using the hot-plate test during the administration of CDDP.

### Experiment II: Influence of CDDP dosing times on adverse effects

CDDP was administered i.v. at 5:00 or 17:00 every 7 days for 4 weeks (*n* = 10). Saline was given to the control group (*n* = 6–12). Body weights were recorded on days 0, 7, 14, 21, and 28 after the first administration of CDDP. Change rates in body weights were calculated as percent body weight change in each rat from the initial value (day 0).

Blood samples were obtained from the tail vein 5, 12, 19, and 26 days after the administration of CDDP in order to measure blood urea nitrogen (BUN) concentrations. All blood samples were immediately centrifuged at 3000 × g for 10 min at 15 °C, and serum was then frozen at −80 °C until assays were performed. BUN concentrations were measured using a manufactured kit (Wako Pure Chemical Industries, Ltd., Japan).

### Experiment III: Influence of CDDP dosing times on recovery from mechanical allodynia after the single administration of CDDP

CDDP was administered i.v. at 5:00 or 17:00 (*n* = 8), and saline was given to the control group (*n* = 8). The von-Frey test was performed on days −7, −5, −3, −1, 1, 2, 3, 6, 10, 13, 17, and 20 before and after the initial administration of CDDP to measure the paw withdrawal threshold. The hot-plate test was performed on day 24 after the administration of CDDP to measure withdrawal latency by heat stimulation.

### Experiment IV: Influence of CDDP dosing times on peripheral neuropathy during its repeated administration

CDDP was administered i.v. at 5:00 or 17:00 every 7 days for 4 weeks (*n* = 4–10). Saline was given to the control group (*n* = 4–12). The von-Frey test was performed on days −7, −5, −3, −1, 3, 6, 10, 13, 17, 20, 24, and 27 before and after the initial administration of CDDP to measure the paw withdrawal threshold. The hot-plate test was performed on days 6 and 28 after the administration of CDDP to measure withdrawal latency by heat stimulation.

### Experiment V: Chronopharmacokinetics of CDDP

CDDP was administered i.v. at 5:00 or 17:00 every 7 days for 4 weeks (*n* = 6). Blood samples were obtained from the tail vein at 5, 15, 30, 60, 120, 240, 360, 480, and 720 min after the first and fourth administration of CDDP. All blood samples were centrifuged immediately at 3000 × g for 10 min at 15 °C. Lumber 4 (L4), L5, and L6 DRG samples were isolated from each anesthetized rats at 24 h after the first and fourth administration of CDDP. Serum and DRG samples were frozen at −80 °C until analyzed.

In order to determine serum CDDP concentrations, 10 μL of rat serum was mixed with 990 μL of 60 % HNO_3_, with Indium ICP-MS standard (Wako Pure chemical Industries, Ltd.) as an internal standard. Each DRG was mixed with 2000 μL of 60 % HNO_3_, with Indium ICP-MS standard as an internal standard, to determine total CDDP concentrations in DRG. This solution was incubated at 100 °C for 30 min, and then diluted 100 times with ultrapure water.

Total platinum concentrations were assayed using inductively coupled plasma mass spectrometry on an ELEMENT2™ ICP-MS (Thermo Fisher Scientific, Inc.). The operating parameters of the ICP-MS instrument were as follows: RF power 1280 W, cool gas flow 15.58 L/min, auxiliary gas flow 0.80 L/min, sample gas flow 0.98 L/min, dead time 25 ns, take-up time 1 min, and four replicates per sample.

### von-Frey test

We used the method of previous report [33]. Mechanical allodynia was assessed using a Touch-test sensory evaluater (Muromachi Kikai Co., Ltd., Japan) at 12:30–13:30 on each measuring day. Each rat was placed in a plastic cage with a wire mesh floor and allowed to acclimate for 10 min before measuring hind paw mechanical thresholds. Filaments, with bending forces that ranged from 1 to 180 g, were applied to the middle of the plantar surface of the right hind paw and held for 5 sec. The withdrawal threshold of the right hind paw was determined by increasing the stimulus strength from the 1 g filament until paw withdrawal occurred.

### Hot-plate test

Thermal hyperalgesia and hypoalgesia were assessed using a hot plate analgesia meter (Muromachi Kikai Co., Ltd., Japan) at 12:30–13:30 on each measuring day. Each rat was placed in a plastic cage with a wire mesh floor and allowed to acclimate for 10 min before measuring hind paw thermal thresholds. A hot plate was pre-heated and maintained at a temperature of 50 ± 0.5 °C. The time for the first sign of nociception, paw licking, flinching, or a jumping response to avoid the heat was recorded. A cut-off period of 60 sec was maintained to avoid damage to the hind paws [34].

### Statistical analysis

All values were shown as a mean with standard error of the mean (S.E.M.). A one-way analysis of variance (ANOVA) was used for multiple comparisons, and Scheffe’s test was used for comparisons between two groups. The Student’s *t*-test was used for comparisons between two groups. *P* values of less than 0.05 were considered significant.

## Results

### Influence the repeated administration of CDDP on peripheral neuropathy

On day 6, the paw withdrawal threshold in the von-Frey test and the withdrawal latency in the hot-plate test were significantly lower in the CDDP-treated group than in the control group (*P* < 0.01, respectively, Table 1). On other days, no significant differences were observed in the paw withdrawal thresholds between the control and CDDP-treated groups. Withdrawal latency was significantly longer in the CDDP-treated group than in the control group on day 27 (*P* < 0.05).

**Table 1:** Time courses of paw withdrawal thresholds and withdrawal latency during the weekly administration of CDDP to rats

Behavioral test	Groups	Time before/after the first administration (days)				
		−1	6	13	20	27
von-Frey test	Control	51.5 ± 5.6 g	51.5 ± 5.6 g	47.3 ± 6.2 g	45.9 ± 7.0 g	51.5 ± 5.6 g
	CDDP	55.8 ± 4.3 g	15.9 ± 2.4 g**	40.3 ± 7.6 g	56.5 ± 8.3 g	70.8 ± 9.5 g
Hot-plate test	Control	15.9 ± 1.5 sec	14.5 ± 0.4 sec	-	-	14.4 ± 1.4 sec
	CDDP	14.4 ± 1.8 sec	9.7 ± 0.9 sec**	-	-	37.1 ± 7.3 sec*

Each data represent the mean with S.E.M. in the von-Frey test (*n* = 8) and in the hot-plate test (*n* = 4), *: *P* < 0.05, and **: *P* < 0.01 using the Student’s *t*-test

### Influence of CDDP dosing times on body weights and BUN

**Fig. 1:**
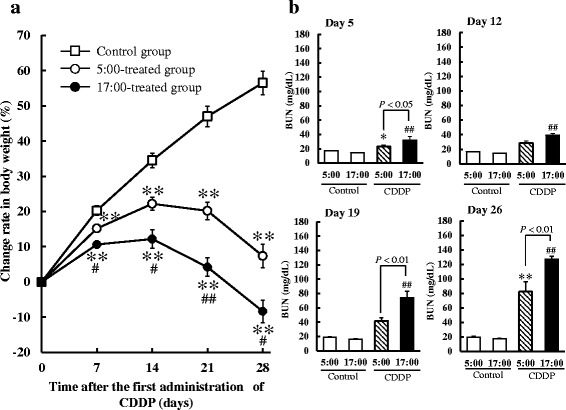
Influence of CDDP dosing times on adverse effects (**a** Change rate in body weight, **b** BUN) in rats. CDDP was administered every 7 days at 5:00 or 17:00. Saline was administered to the control group. Each value represents the mean with S.E.M. (**a** control group (*n* = 12); each CDDP-treated group (*n* = 10). **b** each control group (*n* = 6); each CDDP-treated group (*n* = 10)). **a** **: *P* < 0.01 versus the control group; #*: P* < 0.05 and ##*: P* < 0.01 versus the 5:00-treated group using Scheffe’s test. **b** *: *P* < 0.05 and **: *P* < 0.01 versus the control group at 5:00; #: *P* < 0.05 and ##: *P* < 0.01 versus the control group at 17:00 using Scheffe’s test

Body weights were significantly lower in the CDDP-treated groups than in the control group throughout this study (*P* < 0.01, respectively, Fig. 1a). Decreases in change rates in body weights were significantly better in the 5:00-treated group than in the 17:00-treated group at all points measured (*P* < 0.05 or *P* < 0.01, respectively).

When CDDP was administered every 7 days, BUN levels in the CDDP-treated groups over time were higher than the control levels (Fig. 1b). Increases in BUN concentrations were significantly lower in the 5:00-treated group than in the 17:00-treated group on days 5, 19, and 26 (*P* < 0.05 or *P* < 0.01, respectively).

### Influence of CDDP dosing times on recovery from mechanical allodynia after single administration

**Fig. 2:**
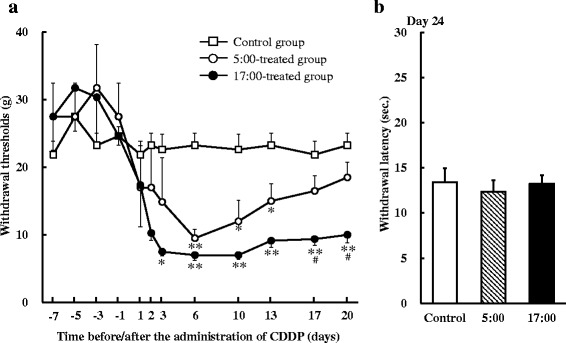
Influence of CDDP dosing times on recovery from mechanical allodynia in rats after a single dose (**a** von-Frey test, **b** Hot plate test on day 24). CDDP was administered to rats at 5:00 or 17:00. Saline was administered to the control group. Each value represents the mean with S.E.M. (*n* = 8). *: *P* < 0.05, **: *P* < 0.01 versus the control group; #: *P* < 0.05 versus the 5:00-treated group using Scheffe’s test

On days 6, 10, and 13 after the single administration of CDDP, paw withdrawal thresholds in the CDDP-treated groups were significantly lower than those in the control group (*P* < 0.05 and *P* < 0.01, respectively, Fig. 2a). The 17:00-treated group continued to maintain mechanical allodynia for 20 days, and paw withdrawal thresholds were significantly lower than those in the control group (*P* < 0.05 and *P* < 0.01, respectively). On the other hand, the paw withdrawal threshold in the 5:00-treated group increased with the passage of time from 10 days after the administration of CDDP. Although paw withdrawal thresholds were significantly higher in the 5:00-treated group than in the 17:00-treated group on days 17, and 20 (*P* < 0.05, respectively), no significant differences were observed between the control and 5:00-treated groups on days 17 and 20.

On day 24, no significant differences were noted in the withdrawal latency by heat stimulation among any groups (Fig. 2b).

### Influence of CDDP dosing times on paw withdrawal thresholds during repeated administration

**Fig. 3:**
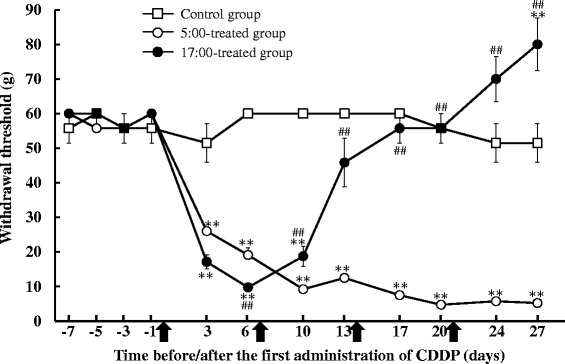
Influence of CDDP dosing times on withdrawal thresholds during its administration to rats. CDDP was administered every 7 days at 5:00 or 17:00. Saline was administered to the control group. Each value represents the mean with S.E.M. (*n* = 8). **: *P* < 0.01 versus the control group; ##: *P* < 0.01 versus the 5:00-treated group using Scheffe’s test

Paw withdrawal thresholds in the 5:00-treated group markedly decreased from normal levels after the first administration of CDDP, and were maintained at low levels throughout the experiment (Fig. 3). Paw withdrawal thresholds were significantly lower in the 5:00-treated group than in the control group throughout this study (*P* < 0.01, respectively). On the other hand, the 17:00-treated group showed temporary decreases in paw withdrawal thresholds, which were significantly lower than those in the control and 5:00-treated groups on day 6 (*P* < 0.01). Paw withdrawal thresholds in the 17:00-treated group increased after the second administration of CDDP (day 7), and were similar to those in the control group by day 17. On day 27, paw withdrawal thresholds were significantly higher in the 17:00-treated group than in the control group (*P* < 0.01). Paw withdrawal threshold were significantly lower in the 5:00-treated group than in the 17:00-treated group from day 10 to 27 (*P* < 0.01, respectively).

### Influence of CDDP dosing times on hypoalgesia during repeated administration

**Fig. 4:**
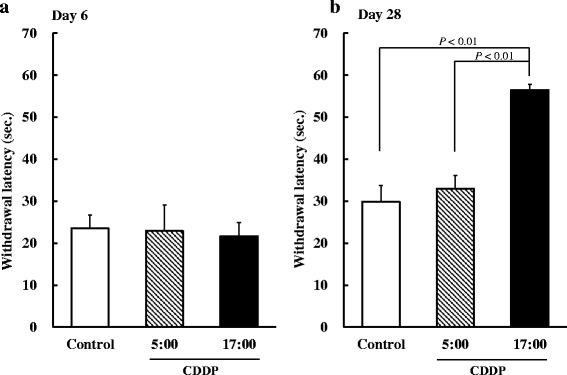
Influence of CDDP dosing times on hypoalgesia during its repeated administration to rats. CDDP was administered every 7 days at 5:00 or 17:00. Saline was administered to the control group. Each value represents the mean with S.E.M. **a** (*n* = 4); **b** (*n* = 8). *P* < 0.01 using Scheffe’s test

No significant differences were observed in the withdrawal latency by heat stimulation among any groups on day 6 after the first administration of CDDP (Fig. 4a). On day 28, the withdrawal latency was significantly higher in the 17:00-treated group than in the control and 5:00-treated groups after the fourth administration of CDDP (*P* < 0.01, respectively, Fig. 4b). On the other hand, no significant differences were observed in the withdrawal latency between the control and 5:00-treated groups.

### Influence of CDDP dosing times on pharmacokinetics in serum and dorsal root ganglia (DRG)

No significant differences in CDDP concentrations were observed in serum between the 5:00 and 17:00-treated groups after the first administration of CDDP (Table 2). Serum concentrations were significantly lower in the 5:00-treated group than in the 17:00-treated group 60, 120, 240, 360, and 720 min after the fourth administration of CDDP (*P* < 0.05 and *P* < 0.01, respectively).

**Table 2:** Influence of dosing time on serum CDDP concentrations after the first or fourth administration of CDDP to rats

Groups	CDDP concentration (μg/mL)								
	5 min	15 min	30 min	60 min	120 min	240 min	360 min	480 min	720 min
The first administration									
5:00	4.62 ± 0.49	3.58 ± 0.17	1.90 ± 0.13	0.87 ± 0.07	0.72 ± 0.03	0.65 ± 0.05	0.63 ± 0.02	0.60 ± 0.01	0.52 ± 0.02
17:00	4.84 ± 0.44	3.68 ± 0.26	1.93 ± 0.15	0.86 ± 0.05	0.71 ± 0.06	0.66 ± 0.04	0.61 ± 0.03	0.57 ± 0.03	0.52 ± 0.05
The fourth administration									
5:00	6.75 ± 0.59	5.34 ± 0.27	3.79 ± 0.20	2.71 ± 0.20	1.74 ± 0.10	1.52 ± 0.13	1.34 ± 0.11	1.12 ± 0.09	1.12 ± 0.04
17:00	7.38 ± 0.24	5.13 ± 0.21	3.81 ± 0.10	2.14 ± 0.09*	1.32 ± 0.05**	1.16 ± 0.06*	1.03 ± 0.07*	0.97 ± 0.06	0.89 ± 0.04**

Each value represents the mean with S.E.M. (*n* = 6), *: *P* < 0.05, and **: *P* < 0.01 using the Student’s *t*-test

**Fig. 5:**
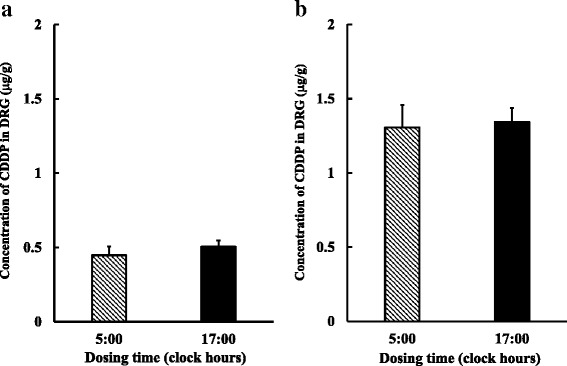
Influence of CDDP dosing times on its concentration in the dorsal root ganglia (DRG) after its first (**a**) or fourth (**b**) administration. CDDP was administered every 7 days at 5:00 or 17:00. Each value represents the mean with S.E.M. (*n* = 6)

No significant differences were observed in CDDP concentrations in DRG between the 5:00 and 17:00-treated groups after its first and fourth administration (Fig. 5).

## Discussion

CDDP-induced peripheral neuropathy is a dose-limiting factor [7]. Although previous studies have attempted to prevent and decrease peripheral neuropathy, effective treatments have not yet been developed [17–20]. CDDP-induced peripheral neuropathy exhibits contrary pain reactions, such as allodynia and hypoalgesia [21–23]. In the early stage, neuropathy appears as allodynia, which is pain induced by a nonnoxious stimulus, and gradually progresses to hypoalgesia, namely, a decrease or the disappearance of sensations by nonnoxious and noxious stimuli, when CDDP is continuously administered [23, 35]. However, it was not reported that an animal model of the CDDP-induced peripheral neuropathy matched clinical situation. Then, at the first, we studied to make the model. When CDDP (4 mg/kg) was given i.v. once weekly, rats showed mechanical allodynia on day 6 after the first administration and prolonged the withdrawal latency by heat stimuli on 6 days (day 27) after the last dose (Table 1). The acute response concerned with the result in mice treated CDDP (2.3 mg/kg) in the past report [36]. This observation that the peripheral neuropathy caused by CDDP gradually progressed to hypoalgesia during the continuous administration, mirrors previous work in rats by Han FY et al. [37]. It was thought that this change in symptom of peripheral neuropathy may reflect the clinical situation.

Peripheral neuropathy induced by oxaliplatin was previously shown to be ameliorated by chronotherapy in patients with measurable metastases from colorectal cancer [38]. Moreover, the chronopharmacology of CDDP had shown many evidences in animal and human [29, 31, 32]. In animal studies, the toxicities such as nephrotoxicity and toxic death were the highest at rest phase in a day. Therefore, we selected the two dosing times (5:00 and 17:00) by referring these reports. In the present study, the group treated with CDDP at rest phase (17:00) showed severe toxicities compared with that at active phase (5:00). The dosing time-dependent differences in adverse effects were consistent with previous findings [28–31]. In the present study, the 17:00-treated group showed 73.9 mg/dL of BUN level on day 19 and 55.8 g of withdrawal threshold on day 20 when CDDP was weekly given. On the other hands, withdrawal threshold in the 5:00-treated group, in which BUN level showed 82.4 mg/dL on day 26, was 5.3 g on day 27. There is no correlation between degree of the nephrotoxicity and the neuropathy. Therefore, we considered that the dosing-time dependency of severity of neuropathy did not necessarily coincide with the CDDP-related toxicities.

The CDDP-treated groups showed significantly lower withdrawal thresholds than the control group immediately after administration of CDDP. However, mechanical allodynia was temporary in the 5:00-treated group after a single administration and the 17:00-treated group when CDDP was given once weekly. The both dosing groups showed exactly the opposite response against mechanical allodynia by the difference in the number of doses. The reason is thought two possible. The neuropathy was improved by repair factor. Or the rats stopped responding to stimuli by progressing hypoalgesia. After a single administration of CDDP, there were no significant differences to heat stimulation among the all groups on day 24. On the other hand, the withdrawal latency was approximately 2-fold longer in the 17:00-treated group than in the control and 5:00-treated groups on day 28 after the repeated administration. Because the increase in withdrawal latency shows the onset of hypoalgesia, increase in withdrawal thresholds after the single dose may indicate recovery from mechanical allodynia in the 5:00-treated group. In the present study, peripheral neuropathy in the 17:00-treated group progressed from allodynia to hypoalgesia, similar to clinical situations [23]. The 5:00-treated group maintained allodynia while CDDP was repeatedly administered. Animal studies previously demonstrated that CDDP induced mechanical allodynia after its administration, and recovery from mechanical allodynia was observed after discontinuation of the treatment [39, 40]. These results suggested that the development of severe CDDP-induced peripheral neuropathy was delayed or inhibited in the 5:00-treated group. Therefore, the selection of an optimal dosing-time may lead to an effective approach for peripheral neuropathy induced by CDDP.

A previous study reported that sensory nerve conduction velocity (SNCV) was decreased by CDDP-induced peripheral neuropathy [41–43]. In order to clarify dosing time-dependent differences in the phenotypes of pain responses, the SNCV of the coccygeal nerve was measured 7 days (day 28) after the fourth administration of CDDP. SNCV was markedly lower in the 17:00-treated group than in the control and 5:00-treated groups (Additional file 1). However, no nerve activity against the electric stimulus was observed in some rats treated with CDDP at 17:00, and thus, SNCV could not be sufficiently calculated. Although it remains unclear whether this result was due to a decline in the conduction velocity because of severe peripheral neuropathy or a technical error, SNCV was markedly decreased in the 17:00-treated group, which showed an increase in the withdrawal latency by the heat stimulation. These responses were observed when the peripheral nerve was injured. We then performed a pathological assessment of the peripheral nerve in the 17:00-treated group 6 days (day 27) after the fourth administration of CDDP in a preliminary study. No marked difference was noted between the control and 17:00-treated groups (Additional file 2). Gilardini A et al. reported that the absence of structural injuries with CDDP-induced peripheral neuropathy [44]. These findings suggested that peripheral neuropathy was more severe in the 17:00-treated group than in the 5:00-treated group; however, pathological assessments could not clarify dosing time-dependent differences in CDDP-induced peripheral neuropathy. Therefore, there was no effective scale to assess histological changes in peripheral nerves such as the sciatic nerve. We intend to investigate genes and proteins related to pain.

The accumulation of CDDP in the DRGs, which contain the cell bodies of primary afferent sensory neurons and play a role in pain mechanisms, is known to induce CDDP-induced peripheral neuropathy [25]. A previous study reported that the enhanced accumulation of CDDP in the DRG positively correlated with the severity of peripheral neuropathy in mice [45]. In the present study, no significant differences were noted in the DRGs levels of CDDP between the 5:00 and 17:00-treated groups. In clinical study, there are few reports of chronopharmacokinetics of CDDP. When CDDP was given at 6:00 or 18:00, the clearance (CL) significantly was higher at 18:00 than at 6:00 [46]. In this study, serum CDDP concentrations were markedly lower in the 17:00-treated group than the 5:00-treated group 60–720 min after the injection. It was consistent with human and animal that disappearance of CDDP from blood was faster in the active phase than the rest phase. However, the toxicities were inhibited in the 5:00-treated group showed higher the serum level. Therefore, we thought that CDDP pharmacokinetics might not contribute to the dosing time-dependent neuropathy. CDDP shows pharmacological effects by crosslinking DNA. It was reported that platinum level in DNA in kidney showed clear dosing time-dependent difference, and the daily variation contributed to degree of nephrotoxicity [47]. In this study, platinum concentration in DNA in DRG was not measured because DRG was small tissue. We are studying measurement method of the platinum level in DNA in nerve tissue including DRG to clarify relevance between the dosing time-dependency of neuropathy and the platinum level in DNA.

Although it is an important to estimate dosing time-dependency of antitumor effect, we have not studied the antitumor effects because LLC-WRC-256 cells, which were breast carcinosarcoma in rat, cannot be transplanted to SD rats. In the preliminary study, the 17:00-treated group (late rest phase) was markedly lower in tumor growth than the control group when CDDP (5 mg/kg) was i.v. given in ICR nu/nu mice bearing A549 cells, which is human lung cancer (Additional file 3). Oxaliplatin, which is the third generation platinum anticancer drug, showed high tumor inhibition rate at early active phase compared with late rest phase [48]. Optimal dosing-time of antitumor effects differs by drugs, cancer cell, and engraftment site. Unlike the side effects that target factors are limited, it is difficult to select a uniform dosage timing for target factor is the variety against cancer. Therefore, we think that long-term CDDP therapy can be carried out by decreasing adverse effects using chronotherapy and anti-tumor effect would be directly or indirectly enhanced as a result.

## Conclusions

In conclusion, pain is an important sign to avoid a variety of risk in life, and hypoalgesia induced by the drug must be avoided. The present study revealed that CDDP-induced peripheral neuropathy which proceeded from allodynia to hypoalgesia was inhibited by the administration of CDDP at a specific time. The adverse effects of CDDP, body weights, BUN levels, and peripheral neuropathy may be improved by identical dosing times. Thus, chronotherapy may contribute to ameliorating adverse effects in patients receiving chemotherapy with CDDP.

## Additional files

Additional file 1: Historogical change of sciatic nerve after the fourth administration of CDDP. No significant differences were observed between the control and 17:00-treated groups. (PPTX 271 kb) Additional file 2: Influence of CDDP dosing times on SNCV after the fourth administration of CDDP to rats on day 27. Each value represents the mean with S.E.M. (*n* = 7–10). (PPTX 74 kb) Additional file 3: Dosing-time dependent change in the antitumor effect after CDDP 5 mg/kg i.v. every 7 days at 5:00 or 17:00 in A549 tumor bearing mice. ●: control group, □: CDDP 5:00 treated group, ■: CDDP 17:00 treated group, Arrows: CDDP administrations. Each value represents the mean ± S.E.M. of 9 or 10 mice. **: *P* < 0.01 (Scheffe’s test). The 17:00 treated group decreased the relative tumor growth compared with the 5:00 treated group. (PPTX 355 kb)

## Abbreviations

ANOVA: Analysis of variance
AUC: Area under the curves
BUN: Blood urea nitrogen
CDDP: *cis*-diamminedichloro-platinum
DRG: Dorsal root ganglia
i.v.: Intravenously
QOL: Quality of life
S.E.M.: Standard error of the mean
SNCV: Sensory nerve conduction velocity
