# Supplementary material for: Influence of dosing times on cisplatin-induced peripheral neuropathy in rats
Source: BMC Cancer. 2016 Sep 27;16:756. doi: 10.1186/s12885-016-2777-0 (PMC5039788; doi:10.1186/s12885-016-2777-0)
Supplement: Additional file 1: — Historogical change of sciatic nerve after the fourth administration of CDDP. No significant differences were observed between the control and 17:00-treated groups. (PPTX 271 kb) [file 12885_2016_2777_MOESM1_ESM.pptx]

## Slide 1
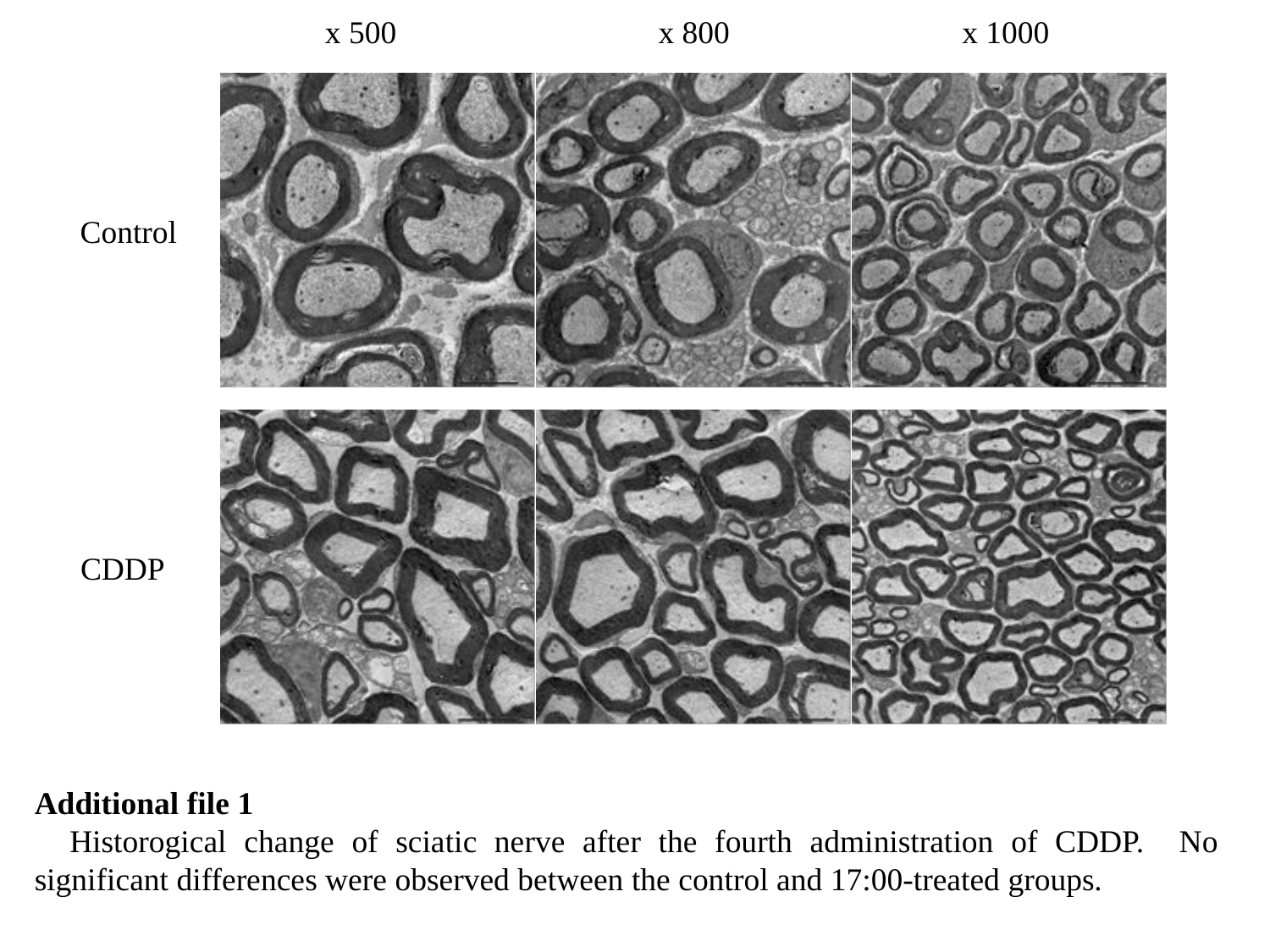

x 500
x 800
x 1000
Control
CDDP
Additional file 1
 Historogical change of sciatic nerve after the fourth administration of CDDP. No significant differences were observed between the control and 17:00-treated groups.
