# Supplementary material for: Influence of dosing times on cisplatin-induced peripheral neuropathy in rats
Source: BMC Cancer. 2016 Sep 27;16:756. doi: 10.1186/s12885-016-2777-0 (PMC5039788; doi:10.1186/s12885-016-2777-0)
Supplement: Additional file 2: — Influence of CDDP dosing times on SNCV after the fourth administration of CDDP to rats on day 27. Each value represents the mean with S.E.M. (n = 7–10). (PPTX 74 kb) [file 12885_2016_2777_MOESM2_ESM.pptx]

## Slide 1
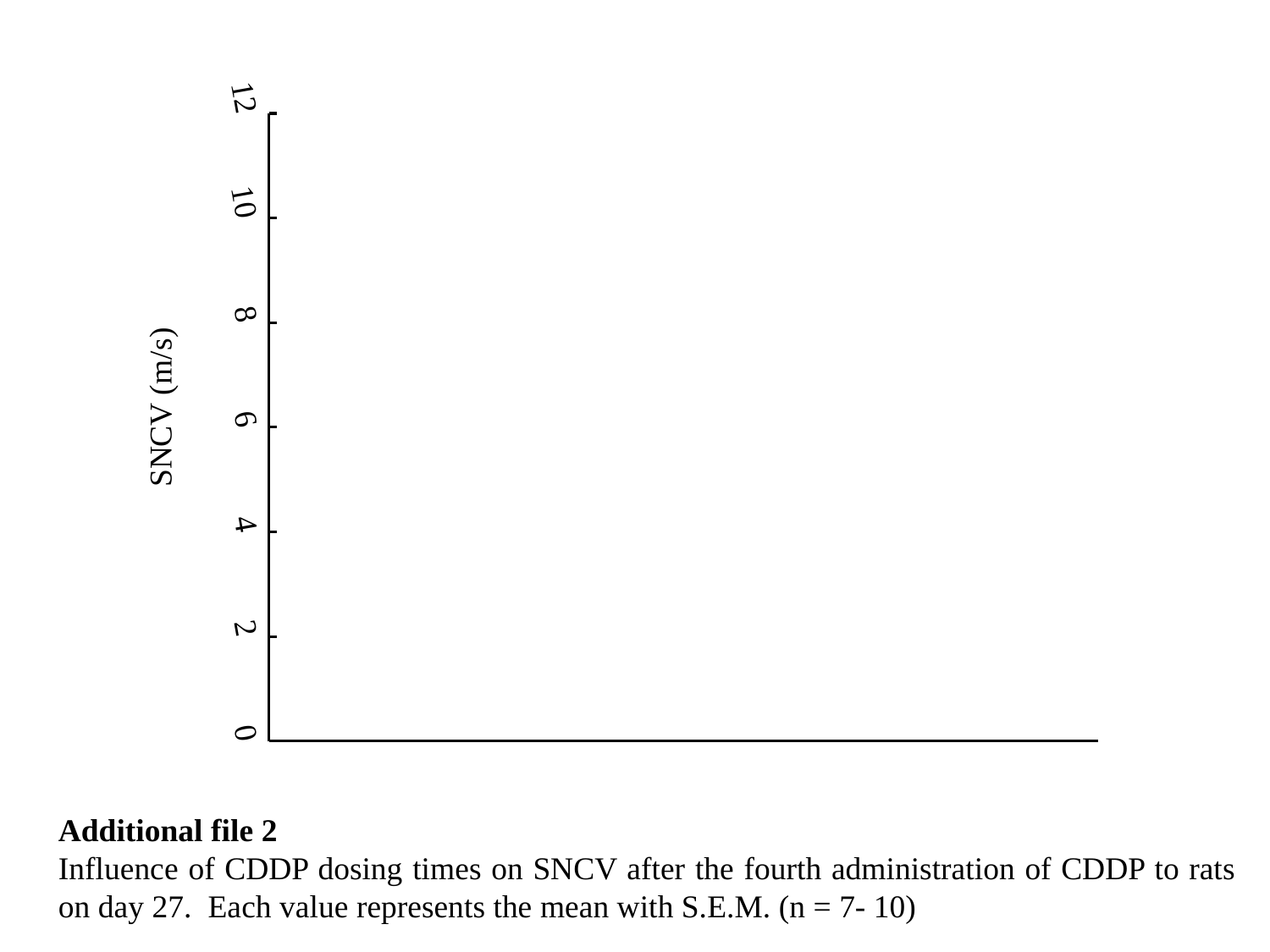

### Chart
| Category | |
|---|---|Additional file 2
Influence of CDDP dosing times on SNCV after the fourth administration of CDDP to rats on day 27. Each value represents the mean with S.E.M. (n = 7- 10)
