# Supplementary material for: Influence of dosing times on cisplatin-induced peripheral neuropathy in rats
Source: BMC Cancer. 2016 Sep 27;16:756. doi: 10.1186/s12885-016-2777-0 (PMC5039788; doi:10.1186/s12885-016-2777-0)
Supplement: Additional file 3: — Dosing-time dependent change in the antitumor effect after CDDP 5 mg/kg i.v. every 7 days at 5:00 or 17:00 in A549 tumor bearing mice. ●: control group, □: CDDP 5:00 treated group, ■: CDDP 17:00 treated group, Arrows: CDDP administrations. Each value represents the mean ± S.E.M. of 9 or 10 mice. **: P < 0.01 (Scheffe’s test). The 17:00 treated group decreased the relative tumor growth compared with the 5:00 treated group. (PPTX 355 kb) [file 12885_2016_2777_MOESM3_ESM.pptx]

## Slide 1
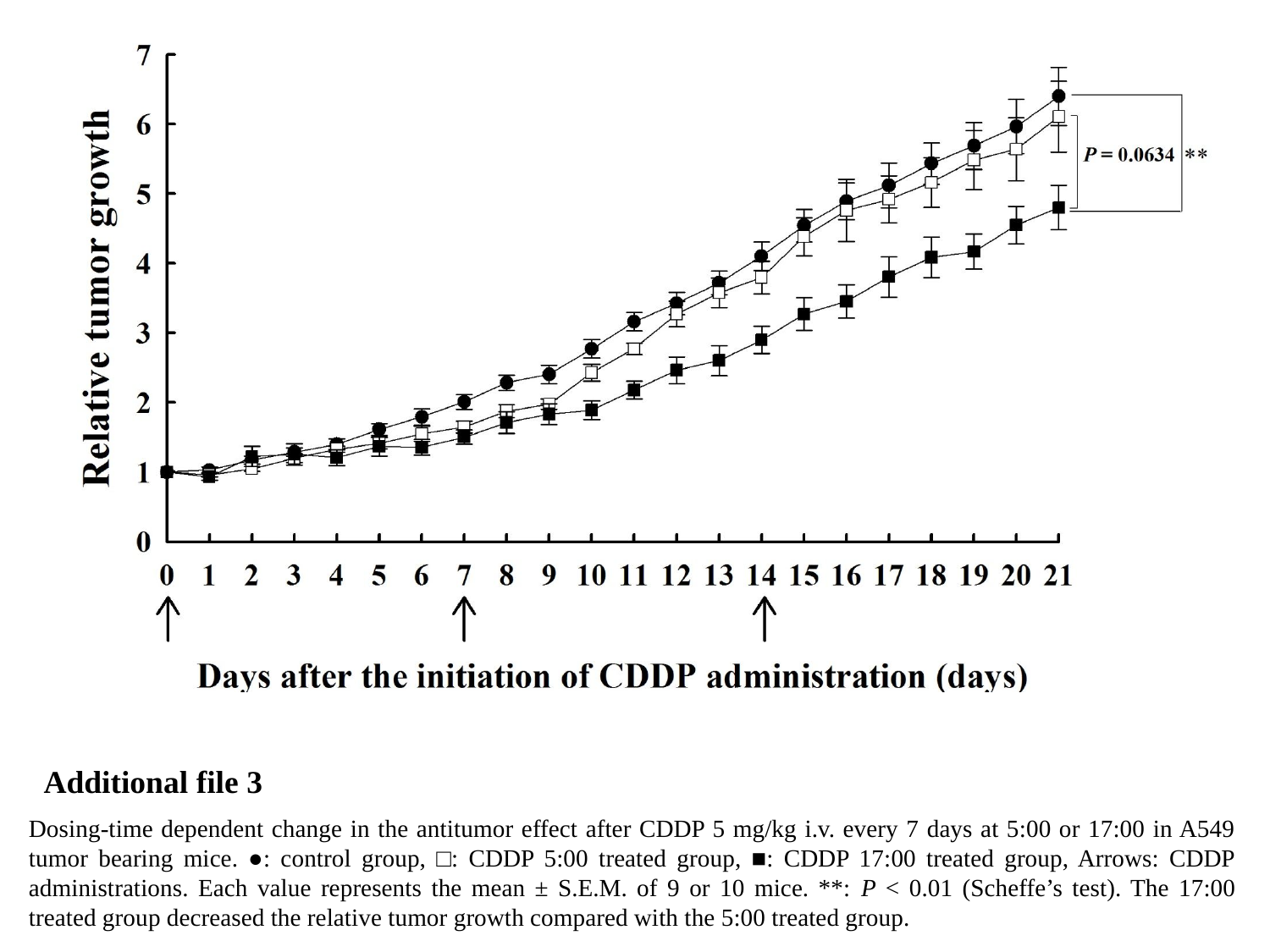

Additional file 3
Dosing-time dependent change in the antitumor effect after CDDP 5 mg/kg i.v. every 7 days at 5:00 or 17:00 in A549 tumor bearing mice. ●: control group, □: CDDP 5:00 treated group, ■: CDDP 17:00 treated group, Arrows: CDDP administrations. Each value represents the mean ± S.E.M. of 9 or 10 mice. **: P < 0.01 (Scheffe’s test). The 17:00 treated group decreased the relative tumor growth compared with the 5:00 treated group.
